# The Effects of Sleeve Gastrectomy on Glucose Metabolism and Glucagon-Like Peptide 1 in Goto-Kakizaki Rats

**DOI:** 10.1155/2018/1082561

**Published:** 2018-02-18

**Authors:** Laiyuan Li, Xiaolin Wang, Liangliang Bai, Huichuan Yu, Zenghong Huang, Anpei Huang, Yanxin Luo, Jianping Wang

**Affiliations:** ^1^Department of Colorectal Surgery, The Sixth Affiliated Hospital of Sun Yat-sen University, Guangzhou, China; ^2^Guangdong Institute of Gastroenterology, Guangdong Provincial Key Laboratory of Colorectal and Pelvic Floor Disease, Guangzhou, China; ^3^Department of Biochemistry and Molecular Medicine, University of California, Davis, Sacramento, CA, USA; ^4^Department of Gastrointestinal Surgery, The First Affiliated Hospital of Sun Yat-sen University, Guangzhou, China

## Abstract

**Purpose:**

To investigate the effects of sleeve gastrectomy (SG) on glucose metabolism and changes in glucagon-like peptide 1 (GLP-1) in Goto-Kakizaki (GK) rats.

**Methods:**

GK rats were randomly assigned to one of three groups: SG, SG pair-fed plus sham surgery (PF-sham), and ad libitum-fed no surgery (control). Food intake, body weight, blood glucose, GLP-1 and insulin levels, and GLP-1 expression in the jejunum and ileum were compared.

**Results:**

The SG rats exhibited lower postoperative food intake, body weight, and fasting glucose than did the control rats (*P* < 0.05). SG significantly improved glucose and insulin tolerance (*P* < 0.05). Plasma GLP-1 levels were higher in SG rats than in control or PF-sham rats in the oral glucose tolerance test (OGTT) (*P* < 0.05). Blood glucose levels expressed as a percentage of baseline were higher in SG rats than in control rats after exendin (9-39) administration (*P* < 0.05). The levels of GLP-1 expression in the jejunum and ileum were higher in SG rats than in PF-sham and control rats (*P* < 0.05).

**Conclusions:**

Improvement of glucose metabolism by SG was associated with increased GLP-1 secretion. SG contributes to an increase in plasma GLP-1 levels via increased GLP-1 expression in the mucosa of the jejunum and/or ileum.

## 1. Introduction

There were 422 million diabetic patients in 2014, and 60% of these patients were obese [1, 2]. Bariatric surgery provides sustained weight loss and glycemic control in obese patients with type 2 diabetes (T2DM), and these effects have been found to be superior to those of conventional therapy in randomized controlled trials [2, 3]. Sleeve gastrectomy (SG) has received increasing attention because of the relatively low rate of complications and the degrees of weight loss and glucose reduction, which are similar to those associated with the common Roux-en-Y gastric bypass (RYGB) [4–6]. SG involves the removal of the greater curvature of the stomach from the angle of His to the distal antrum [7]. Some of the beneficial effects of SG on glycemic homeostasis are secondary to weight loss, but the improvement in glucose control often occurs before substantial weight loss [8, 9]. Furthermore, these changes are not observed after an equivalent amount of diet-induced weight loss [10]. The hindgut hypothesis proposes that the stimulation of the distal ileum with the early arrival of partially digested nutrients is responsible for the improved glucose tolerance after bariatric surgery [11]. The remarkable changes in intestinal hormones, such as glucagon-like peptide 1 (GLP-1), are the basis of this hypothesis [12]. GLP-1 is a 30-amino acid product of the preproglucagon gene, and GLP-1 is converted by proprotein convertase 1 (PC1/3), which is encoded by the proprotein convertase subtilisin/kexin type 1 (PCSK1) gene [13–15]. GLP-1 reduces glucose excursion by inhibiting *β*-cell apoptosis and promoting the proliferation and stimulation of *β*-cell insulin secretion [16, 17]. SG produces a significantly higher resolution rate of T2DM with obesity [18]. Whether the beneficial effect of SG on glucose homeostasis is triggered by undigested nutrients in the ileum or the jejunum remains to be elucidated. This study used nonobese diabetic Goto-Kakizaki (GK) rats to investigate the effects of SG on glucose metabolism and GLP-1 levels in these GK rats.

## 2. Methods

### 2.1. Experimental Animals

Thirty nonobese, diabetic male GK rats (Chang Zhou Cavens Laboratory Animal Ltd., Changzhou, China), 12 weeks old, were individually housed and maintained with a 12 h light/dark cycle (lights off at 20:00 h) at 22–26°C and 40–60% humidity. The rats were acclimatized for two weeks and randomized to the SG, SG pair-fed plus sham surgery (PF-sham), or ad libitum-fed no surgery (control) groups. All applicable institutional and/or national guidelines for the care and use of animals were followed. The Sun Yat-sen University Animal Care and Research Committee approved all the experimental procedures.

### 2.2. Surgical Procedures

Anesthesia was standard among all rats that had either an SG or sham operation. SG was performed as previously described [19]. Briefly, gastric connections to the spleen and liver were released, and vascular clamps were placed along the greater curvature from the antrum to the fundus. With the use of a scalpel, approximately 80% of the total stomach was removed along the vascular clamps. The remnant stomach was sterilized with iodine, and the incision was closed with interrupted Prolene 6-0 sutures (Ningbo Medical Needle Co., Ltd., Ningbo, China). The sham surgery technique (PF-sham rats) involved the same procedure described above, except for the stomach resection. The operative time for the PF-sham rats was prolonged to ensure a degree of anesthesia-related stress equivalent to that in the rats undergoing SG.

### 2.3. Postoperative Care

After surgery, the GK rats were placed on a heated mat until they had fully recovered from anesthesia. All the rats received subcutaneous injections of ceftriaxone (5 mg/100 g, once daily for 3 days) and warm saline (20 ml per day for 3 days) postoperatively. The rats were provided free access to water after 24 h after surgery. Regular chow was resumed three days or even later after surgery. All the PF-sham rats were pair-fed according to the average daily food intake of the SG rats from the previous experiment. The control group rats received food ad libitum.

### 2.4. Body Weight and Food Intake

Body weight and food intake were measured prior to surgery and afterward for five consecutive days and then weekly between 09:00 and 11:00 h during the study period.

### 2.5. Glucose Tolerance Tests

The oral glucose tolerance test (OGTT) and IP injection glucose tolerance test (IPIGTT) were performed at two and seven weeks after surgery, respectively. The rats were fasted for 6 h (from 08:00 to 14:00 h) and then given an oral gavage of 25% glucose (2 g/kg) or an IP injection of 25% glucose (1.5 g/kg). Blood glucose, insulin, and GLP-1 were measured. The area under the curve (AUC) for OGTT (AUC_OGTT_) was calculated starting at 0 min and ending at 120 min for each group.

### 2.6. Insulin Tolerance Tests

Insulin tolerance tests (ITTs) were performed at 3 and 12 weeks after surgery. The rats were fasted for 6 h and were given an IP injection of insulin (0.6 IU/kg). Glucose levels were measured at 0, 30, 60, 90, and 120 min. The ITT result was calculated as the AUC_ITT_.

### 2.7. Intraperitoneal Injection of Exendin (9-39)

The GLP-1 receptor antagonist exendin (9-39) (American Peptide Company, CA; 50 *μ*g/kg) was injected intraperitoneally to evaluate the effect of GLP-1 on glucose and insulin metabolism in the GK rats at six weeks postoperatively. Blood glucose and insulin concentrations were also expressed as a percentage of the baseline glucose values. Nine weeks later, an OGTT was performed after an IP injection of exendin (9-39).

### 2.8. GLP-1 and Insulin Measurements

Blood was collected in tubes containing an antiproteolytic cocktail every 15 min for 1 h. The plasma was separated and stored at −80°C until assayed. The plasma insulin and GLP-1 levels were measured using commercial enzyme-linked immunosorbent assay kits (Millipore, St. Charles, MO) according to the manufacturer's instructions.

### 2.9. Tissue Harvesting

The beginning of the jejunum and the terminal ileum (3 cm) were removed and fully washed with sterile saline solution at 18 weeks postsurgery. Half of the tissue samples of the jejunum and ileum of each rat were fixed in 4% formalin and embedded in paraffin for immunohistochemical processing. The other half of the tissue samples was harvested and immediately frozen in liquid nitrogen and stored at −80°C for RNA extraction.

### 2.10. Histomorphometry and Immunohistochemistry

Tissue sections were stained with hematoxylin and eosin using standard procedures and examined by light microscopy. The villus height and width were recorded from 30 well-oriented villi on each sample slide. Formalin-fixed, paraffin-embedded sections of jejunal and ileal tissues were immunostained using an anti-GLP-1 antibody. Samples with brown-yellow granules under a light microscope were considered positive for GLP-1 expression.

### 2.11. RNA Isolation and Quantitative Real-Time PCR (qRT-PCR)

qRT-PCR was used to examine the relative mRNA levels of preproglucagon and PCSK1 in the jejunal and ileal segments. Total RNA was isolated from the intestinal mucosa using TRIzol (Ambion, Thermo Fisher) and purified with an RNeasy Mini kit (Qiagen, Suzhou Industrial Park, China) according to the manufacturers' protocols. Each RNA sample was converted into cDNA using the ReverTra Ace® qPCR RT Master Mix with gDNA Remover (Toyobo, Osaka, Japan). The quantification of cDNA was performed via qRT-PCR in a LightCycler® 96 System (Roche Applied Science, Indianapolis, IN, USA) with a FastStart Essential DNA Green Master kit (Roche) under the following conditions: 20 *μ*l per reaction, 95°C for 600 s, 45 cycles of 95°C for 10 s, 60°C for 10 s, and 72°C for 10 s.

### 2.12. Statistical Analyses

The results were expressed as the means ± standard deviation (SD). The AUCs for the OGTT and ITTs were calculated through trapezoidal integration. Differences between the groups were analyzed using Student's *t*-test or a one-way analysis of variance (ANOVA) with the Bonferroni post hoc test, when appropriate. All statistical analyses were performed using SPSS version 21.0, and differences with *P* < 0.05 were considered statistically significant.

## 3. Results

### 3.1. SG Reduces Food Intake, Body Weight, and Fasting Glucose Levels

**Figure 1:**
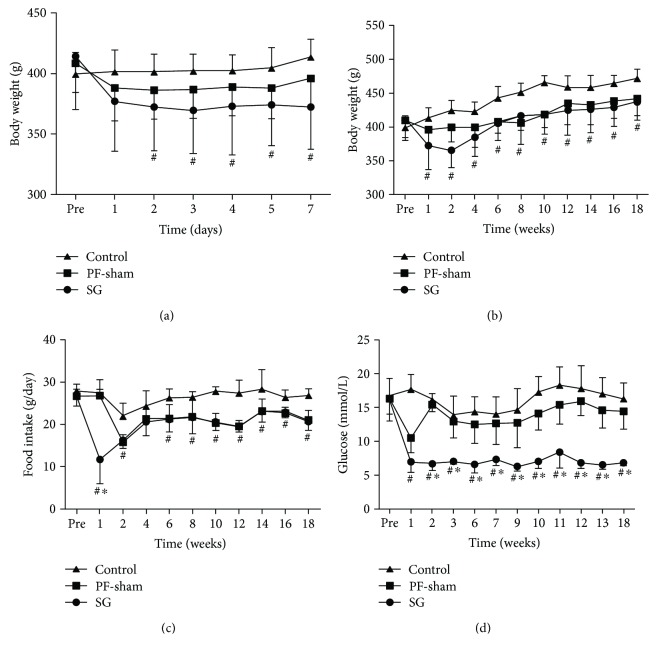
Effects of sleeve gastrectomy on food intake, body weight, and fasting glucose levels in GK rats. Line charts show body weights (a, b), food intake (c), and glucose (d). ^#^*P* < 0.05 versus the controls; ^∗^*P* < 0.05 versus PF-sham.

The mean preoperative food intake, body weight, and fasting glucose level did not differ among the three groups (*P* > 0.05) (Figure 1). The body weights of the SG rats after surgery were lower than those of the control rats (*P* < 0.05) (Figures 1(a) and 1(b)). A significant decrease in food intake was observed in the SG rats compared with the control rats in the first week after surgery. This decrease began to reverse during the second week, but the food intake level of the SG rats was lower than that of the control rats (*P* < 0.05) (Figure 1(c)). Fasting blood glucose was reduced in the SG rats after surgery compared with that in the PF-sham and control rats (*P* < 0.05) (Figure 1(d)). These data suggest that SG reduced food intake, body weight, and fasting glucose levels.

### 3.2. SG Improves Glucose Homeostasis

**Figure 2:**
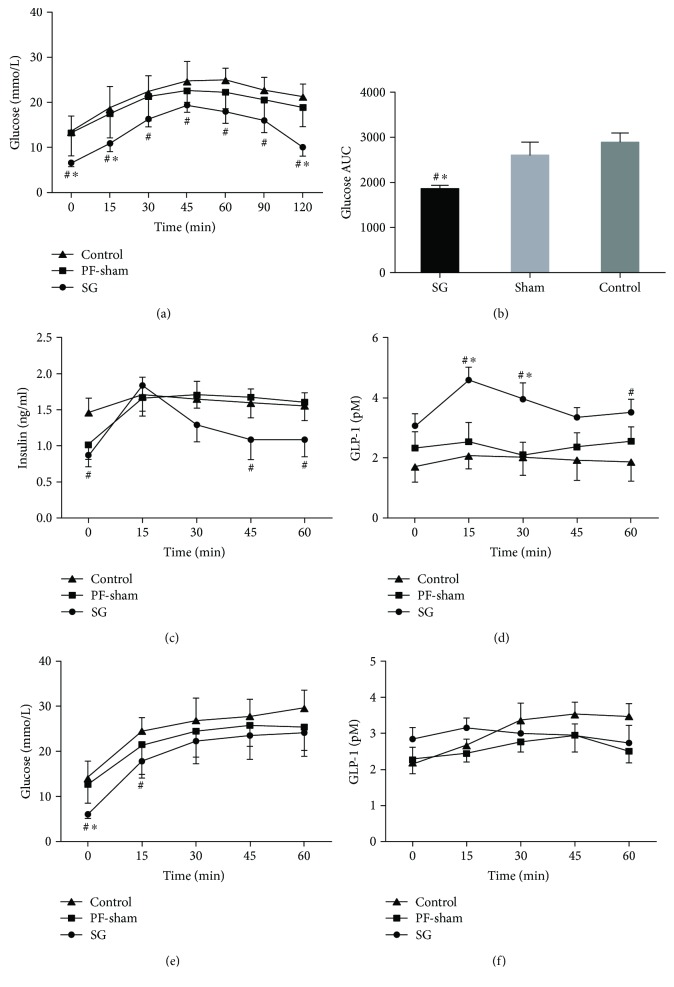
Effects of sleeve gastrectomy on glucose metabolism in GK rats. Line charts or bar graphs show the blood glucose (a), the AUC_OGTT_ (b), insulin (c), and GLP-1 (d) in the oral glucose tolerance test, and blood glucose (e) and GLP-1 (f) after an IP injection of glucose. ^#^*P* < 0.05 versus the controls; ^∗^*P* < 0.05 versus PF-sham.

Blood glucose was lower in the SG rats than in the control rats in the OGTT at 0, 15, 30, 45, 60, 90, and 120 min (*P* < 0.05) (Figure 2(a)). The AUC_OGTT_ of the SG rats was smaller than that of the PF-sham and control rats (*P* < 0.05) (Figure 2(b)). Insulin levels peaked at 15 min in all the groups, but the insulin levels were significantly lower in the SG rats than in the control rats during the OGTT at 0, 45, and 60 min (*P* < 0.05) (Figure 2(c)). GLP-1 levels peaked at 15 min in all the groups, but the peak was not obvious in the PF-sham and control rats. The GLP-1 level in the SG rats was higher than that in the PF-sham or control rats at 15, 30, and 60 min (*P* < 0.05) (Figure 2(d)). In the IPIGTT, the blood glucose levels increased in the three groups, but GLP-1 levels did not increase significantly, and the blood glucose and GLP-1 levels were not different among the three groups at 30, 45, and 60 min (*P* > 0.05) (Figures 2(e) and 2(f)). Together, these data suggest that SG profoundly improved postprandial glucose metabolism in the GK rats, and higher insulin release was observed following increased GLP-1 after the OGTT, but not the IPIGTT, in the GK rats.

### 3.3. SG Improves Insulin Tolerance

**Figure 3:**
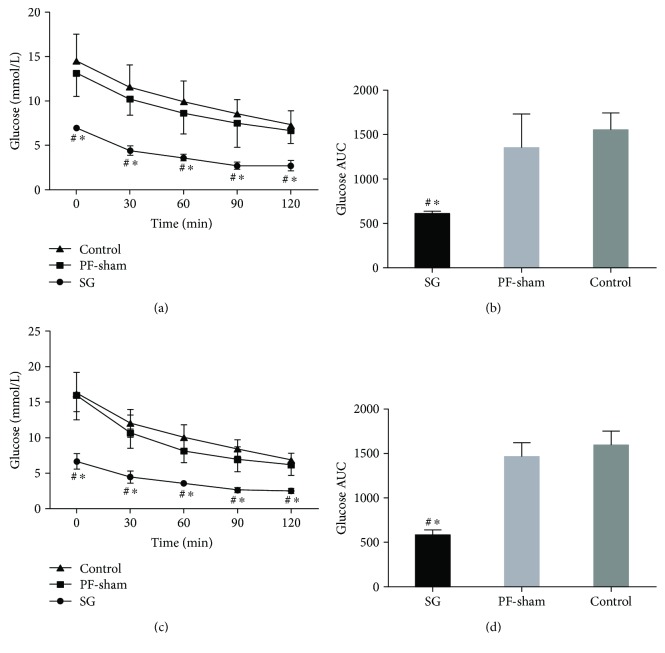
Effects of sleeve gastrectomy on insulin tolerance in GK rats. Line charts show the blood glucose (a) and the AUC_ITT_ (b) at 3 weeks, and the blood glucose (c) and the AUC_ITT_ (d) at 12 weeks after an IP injection of insulin. ^#^*P* < 0.05 versus controls, ^∗^*P* < 0.05 versus PF-sham.

An ITT analysis was performed three weeks after surgery. The blood glucose level was reduced in the SG group compared with the control and/or PF-sham groups at baseline (0) and at 30, 60, 90, and 120 min (Figure 3(a)), and the AUC_ITT_ of the SG rats was smaller than that of the PF-sham and control rats (*P* < 0.05) (Figure 3(b)). An ITT was also performed at 12 weeks. Blood glucose in response to insulin fell to significantly lower levels in the SG rats than in the PF-sham and control rats at 0, 30, 60, 90, and 120 min (*P* < 0.05) (Figure 3(c)). The blood glucose AUC_ITT_ was significantly lower in SG rats compared with PF-sham and control rats (*P* < 0.05) (Figure 3(d)). Together, these data suggest that SG can improve insulin resistance in GK rats.

### 3.4. GLP-1 Stimulates Insulin Secretion

**Figure 4:**
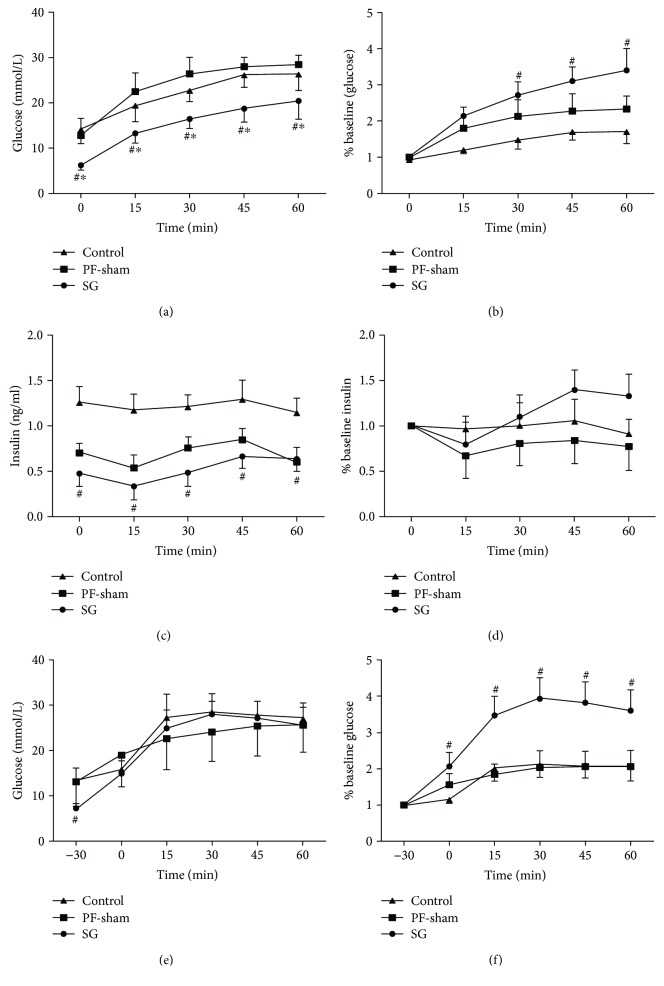
Effect of exendin (9-39) on glucose metabolism in GK rats. Line charts show the blood glucose level (a) expressed as a percentage of the baseline level (b) and insulin level (c) expressed as a percentage of the baseline level (d) after an IP injection of exendin (9-39). Blood glucose levels (e) expressed as a percentage of the baseline level (f) with an IP injection of exendin (9-39), 30 min before the OGTT. ^#^*P* < 0.05 versus the controls; ^∗^*P* < 0.05 versus PF-sham.

Glucose levels increased from 0 to 60 min in all groups after an IP injection of the GLP-1 (7-36) receptor antagonist exendin (9-39). However, the blood glucose level was lower in the SG group than in the control and/or PF-sham groups when expressed as an absolute value (Figure 4(a)) and as a percentage of the baseline values at 30, 45, and 60 min (*P* < 0.05) (Figure 4(b)). A decrease in insulin levels was observed 15 min after exendin (9-39) administration in all the groups. The plasma insulin levels were significantly lower at baseline in the SG rats than in the control rats (*P* < 0.05) (Figure 4(c)), but these levels were not significantly different from those observed in the PF-sham or control rats when expressed as a percentage of baseline at any point (*P* > 0.05) (Figure 4(d)). Blood glucose levels were not significantly different between the SG and control rats after an oral gavage of glucose in the rats pretreated with the exendin (9-39) (Figure 4(e)), but these levels were significantly different when expressed as a percentage of baseline (*P* < 0.05) (Figure 4(f)). These data indicate that GLP-1 reduced blood glucose by promoting insulin release.

### 3.5. SG Stimulates Jejunal and Ileal Villi Growth

**Figure 5:**
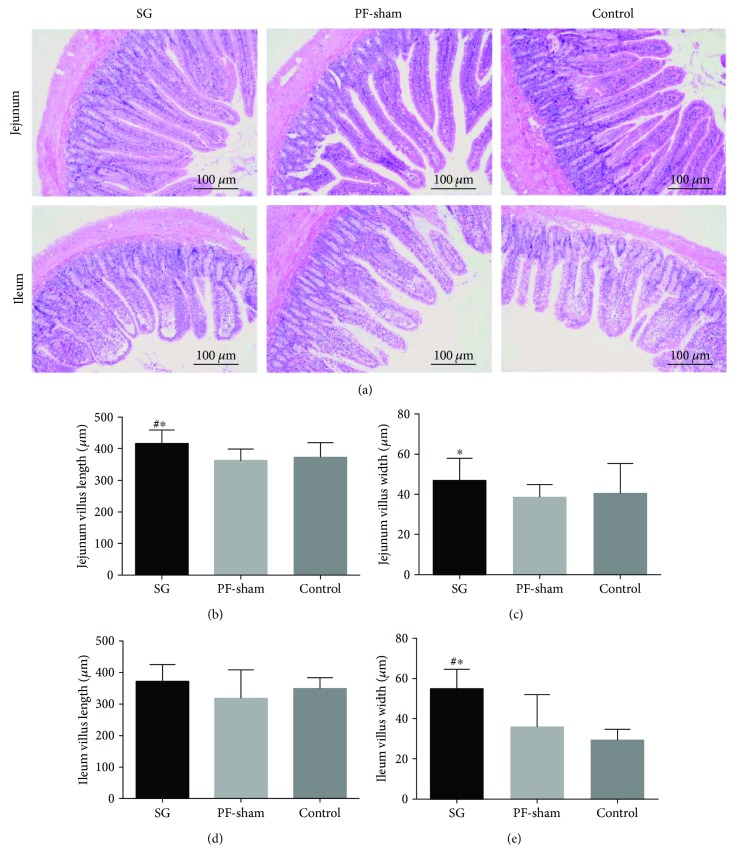
Effects of sleeve gastrectomy on the morphology of the jejunal and ileal segments. (a) Representative images of the jejunal and ileal sections of the GK rats stained with hematoxylin-eosin are shown (magnification 100x; scale bar = 100 *μ*m). Bar graphs show the jejunal villus length (b) and width (c) and the ileal villus length (d) and width (e). ^#^*P* < 0.05 versus the controls; ^∗^*P* < 0.05 versus PF-sham.

Matching sections of jejunum and ileum were collected from the SG, PF-sham, and control rats at the end of the study to assess the effect of SG on intestinal morphology (Figure 5(a)). Increases in villus width in the jejunum and ileum were observed in the SG rats (*P* < 0.05) (Figures 5(c) and 5(e)). There was a significant difference in the length of the jejunal villi between the control and SG rats (*P* < 0.05) (Figure 5(b)), but the ileal villi length was not altered significantly (*P* > 0.05) (Figure 5(d)). These data indicate that SG leads to a profound morphological adaptation in the jejunum and ileum.

### 3.6. SG Increases GLP-1 Expression Levels in the Jejunum and Ileum

**Figure 6:**
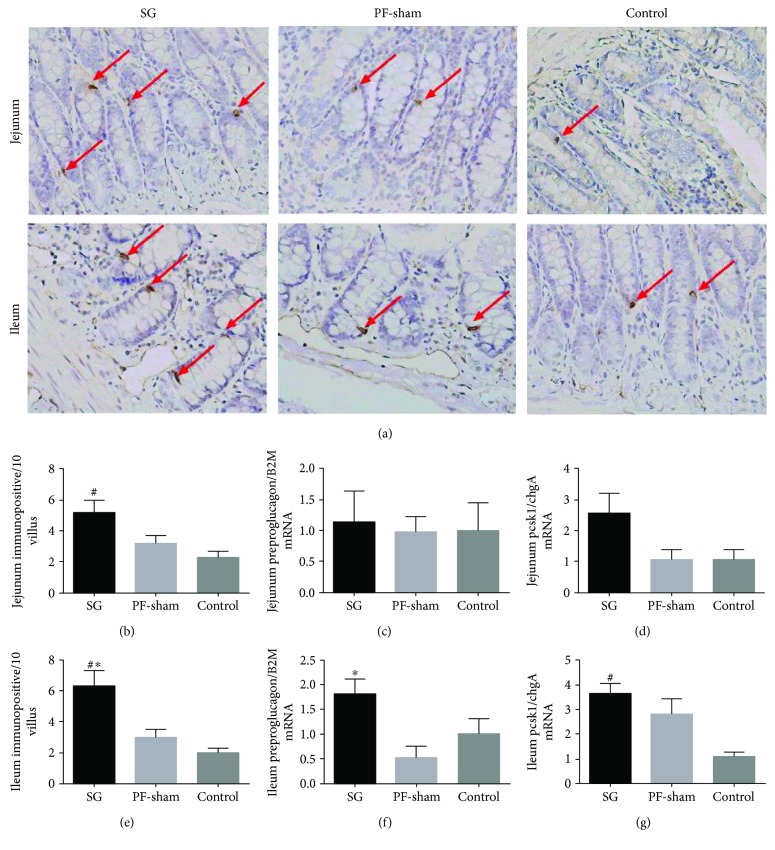
Effects of sleeve gastrectomy on the expression levels of the GLP-1 protein and the preproglucagon and PCSK1 genes in jejunal and ileal segments. Representative micrographs (400x magnification) of GLP-1 immunopositive cells (a) and the relative numbers of GLP-1 immunopositive cells (b, e). Expression levels of the preproglucagon (c, f) and PCSK1 genes (d, g) in the jejunum and ileum are shown. Brown GLP-1-positive staining is indicated by arrows. ^#^*P* < 0.05 versus the controls; ^∗^*P* < 0.05 versus PF-sham.

We investigated GLP-1 protein expression, and preproglucagon and PCSK1 mRNA levels in the jejunum and ileum using immunohistochemistry and qRT-PCR, respectively, to determine the actions of SG in the jejunum and ileum in the GK rats. GLP-1 protein expression was significantly higher in the SG rats than in the PF-sham and control rats (*P* < 0.05) (Figures 6(a), 6(b), and 6(e)). Preproglucagon and PCSK1 mRNA levels in the jejunum were not altered significantly in the SG rats compared with the control rats (*P* > 0.05) (Figures 6(c) and 6(d)), but upregulated PCSK1 mRNA was observed in the ileum (*P* < 0.05) (Figures 6(f) and 6(g)). Together, these data indicate that SG leads to increased GLP-1 expression in the mucosa of the jejunum and ileum.

## 4. Discussion

Recent studies have shown that medical therapy for obesity and comorbid T2DM is not as successful as bariatric surgery [20]. SG is gaining in popularity as an independent procedure because this approach is simple and effective in weight loss and resolves diabetes [21]. However, the underlying mechanisms of the metabolic benefits produced by SG remain largely unknown. This study investigated the effects of SG surgery on T2DM in GK rats. We found that SG increased postprandial GLP-1 levels, and insulin sensitivity, and the effect of improved glucose tolerance after SG was reversed by GLP-1 receptor antagonism. Notably, improvements in glucose metabolism were further supported by the increased expression of GLP-1 in the mucosa of jejunal and ileal tissues, which is consistent with the increased plasma GLP-1 level.

In the present work, we evaluated the effects of SG on body weight and food intake in GK diabetic rats. Our results showed that SG was effective in restraining body weight gain and reducing food intake of GK rats for the 18-week study duration. Basso et al. [22] reported that glandular gastrectomy, leaving the forestomach almost intact, did not induce significant body weight loss or reduced food intake from third week after surgery. The reason is that the aglandular stomach remains intact (the gastric section responsible for food storage and digestion in rat); this may permit normal food intake comparable to that of sham operation rats. Rodriguez et al. [23] have reported that a pair-fed group of rats had higher body weights and more adiposity than did a group of SG rats. However, no differences in body weight were observed between the PF-sham and SG-operated rats in our study. The food intake and body weight of the SG rats in our study were similar to those of the pair-fed rats in both the current study and a previous study [24], but we still detected a markedly reduced fasting glucose level in the SG rats. Although previous studies have demonstrated that glucose metabolism improves in association with weight loss after SG surgery [5, 25], other evidence support the ability of SG to elicit weight-independent changes to glucose homeostasis [22]. These results suggest that SG improves glucose metabolism, probably through weight-independent effects, in the GK rat model.

Our results demonstrated that the SG-treated rats exhibited better glucose and insulin tolerance than did the PF-sham rats with similar food intakes and body weights. This result is consistent with previous findings that SG ameliorates T2DM in GK rats more rapidly and effectively than does a greater restriction of food intake in matched nonoperated rats [26]. Clinical observations have demonstrated that low-calorie diets do not improve diabetes in obese patients who subsequently experience diabetes resolution from RYGB surgery [27]. Patients undergoing biliopancreatic diversion exhibit a temporary food intake reduction, but their eating capacity is restored or increases over time while their blood glucose levels remain under control [28]. SG may not just be a useful bariatric surgery; actually, there are differences in digestive and hormonal changes between SG and the traditional restrictive procedures, vertical banded gastroplasty and adjustable gastric banding (AGB). The hindgut hypothesis suggests that the early presentation of incompletely digested nutrients to the distal intestine after surgery leads to greater GLP-1 secretion from L-cells, subsequently improving insulin action [11]. In the SG rats, we observed a higher level of insulin excursion in the OGTT with the increase in GLP-1 level, but this effect was not observed in the IPIGTT. These results suggest that enhanced insulin release is driven by an enhanced incretin effect, such as that of GLP-1, rather than by the direct actions of circulating glucose on *β*-cells, which is consistent with the hindgut hypothesis. Clinical observations have demonstrated that post-SG hormonal changes occur even on the third postop day and before any food intake [29]. Peterli et al. [30] have found that postprandial plasma insulin is higher in RYGB patients than in SG patients one week postoperatively, but both surgery groups exhibit similar insulin levels at three months. Although there was a correlation between insulin sensitivity and body weight, the major driver of the improvement in insulin sensitivity after SG was GLP-1 secretion [31]. Changes in GLP-1 are often considered a mechanism for the weight-independent effects of bariatric surgery on glucose homeostasis, which may explain why purely restrictive procedures or nonsurgical weight loss have little effect on glucose control [32]. Gastaldelli et al. [33] studied obese patients without diabetes and demonstrated a difference in insulin sensitivity between the AGB and RYGB; specifically, they note an improvement in insulin sensitivity of adipose and muscle tissue in the RYGB. To assess the degree to which increased GLP-1 release in SG rats improved oral glucose tolerance, we also administered exendin (9-39) to block the GLP-1 receptor in the GK rats. We found that the majority of the incretin effect after SG was prevented by blocking GLP-1 receptors. Other studies have also demonstrated worse glucose control with GLP-1R blockade in rats after duodenojejunal exclusion, ileal translocation, and gastric bypass surgeries [5, 34, 35]. The clinical data also support the hypothesis that changes in the pattern of GLP-1 secretion mediate the effect of SG on diabetes [36]. Together, our results provide evidence that the improvement of glucose tolerance following SG surgery is mediated by enhanced GLP-1 action.

It is commonly believed that increased postprandial GLP-1 levels after RYGB are a result of gastrointestinal rearrangement, which produces a shortcut for undigested nutrients to enter the distal gut, thereby stimulating GLP-1-producing L-cells [5, 37]. However, the number of L-cells increases only in limbs that are exposed to undigested nutrients, the common and Roux limbs, but not the biliopancreatic limb, suggesting that the stimulation is a direct consequence of abnormal perfusion with undigested nutrients. SG does not involve a shortcut for nutrients, but similar increases in plasma GLP-1 levels have been observed [5]. The increases in intestinal villus length and width confirm the induction of adaptations, suggesting that there are significant effects of local nutrient contact in the stimulation of adaptations after SG in our study. Therefore, we further detected the protein expression levels of GLP-1 in the jejunal and ileal segments and found that the level of GLP-1 protein expression in the intestinal specimens was increased, which is consistent with the increased plasma GLP-1 level in the GK rats after SG. Wang et al. [38] have presented similar conclusions in the jejuno-ileal circuit procedure. We further investigated GLP-1-related gene and find that the preproglucagon and PCSK1 mRNA levels were increased in the ileal mucosa of the SG rats. Patriti et al. [12] indicated that good glycemic control, higher proglucagon mRNA expression has been shown to be present in the transposed ileal segment. We only examined PCSK1; therefore, we cannot rule out that other rate-limiting enzymes of GLP-1 were altered. Taken together, our results demonstrated that the level of GLP-1 expression not only in the ileal mucosa but potentially also in the jejunal mucosa was increased. These analyses suggest that SG resulted in stimulated intestinal villus growth and improved jejunal and/or ileal tissue expression of GLP-1, which is consistent with the increased plasma concentrations of GLP-1. The present study is, to our knowledge, the first report on the protein and mRNA expression levels of GLP-1 of the jejunal mucosa in SG procedure using the GK rat model. Gastric emptying after SG is accelerated for liquids or solids in the majority of obese patients [39]. Unfortunately, a cause-effect relationship between SG and gastric emptying was not confirmed in our study. However, other studies found that SG does not alter gastric emptying in obese patients [40, 41]. This result may have been due to differences in the size of the small pouch that impairs gastric emptying, differences in surgical technique, or a failure to distinguish between nondiabetics and diabetics in obese patients, given that diabetes may also affect gastrointestinal motility [42]. Understanding the role of SG in the GK rat model may provide further insight into surgically induced improvements in metabolism and may lead to the development of less invasive surgeries for obesity-related diabetes.

In conclusion, SG improved glucose metabolism, and insulin release was associated with increased GLP-1 secretion. SG contributes to an increase in plasma GLP-1 levels via increased GLP-1 expression in the mucosa of the jejunum and/or ileum. The effect of improved glucose tolerance after SG was reversed by GLP-1 receptor antagonism. This study provides evidence that the improvement of glucose metabolism following SG surgery is mediated by enhanced GLP-1 action.

Certain limitations must be considered in the interpretation of our results. First, a cause-effect relationship between SG and gastric emptying was not confirmed in our study. Second, our experiments used a nonobese GK rat model; future studies should utilize an obese model to obtain results more pertinent to human patient populations.

## Additional Points

*Animal Rights.* All applicable institutional and/or national guidelines for the care and use of animals were followed.
